# The 2,4,6,8‐Tetramethylhomotropyliumdication

**DOI:** 10.1002/jcc.70218

**Published:** 2025-08-20

**Authors:** Matthias Bremer

**Affiliations:** ^1^ Justus‐Liebig‐Universität, Institut für Organische Chemie Giessen Germany

## Abstract

In 1976 George A. Olah et al. synthesized the Hückel‐aromatic 1,3,5,7‐tetramethylcyclo‐octatetraene dication **3** under stable ion conditions. As observed by NMR spectroscopy, **3** rearranges at −20°C to the 1,3,5,7‐tetramethylbicyclo[3.3.0]‐dication **5**. This is an unexpected result that has not been commented upon in the original papers or considered in the literature for half a century. We propose a mechanism for the rearrangement of **3** to **5** and discuss the relative instability of the latter with respect to the isomeric 2,4,6,8‐tetramethylbicyclo[3.3.0]dication **6**. The unknown isomeric dication **6** is predicted to be 34.4 kcal mol^−1^ lower in energy than **5** and 40.9 kcal mol^−1^ lower in energy than **3**.

## 1 Introduction

The first year of the new millennium marked the centennial of research on carbocations. George A. Olah, the preeminent researcher in the field and recipient of the 1994 Nobel prize, published a comprehensive perspective on the occasion [1]. While the publication flood of the 1960s–1980s has long subsided, carbocation chemistry retains its undisputed didactic value for the understanding of reaction mechanisms and is fundamental for the structural and mechanistic chemistry of electron deficient compounds.

Several of the “old‐fashioned” (the term “classical” [2] is misleading in this context) carbocations and dications have been revisited in the 21st century, highlights being papers on the notorious 2‐norbornyl cation **1** [3, 4] and Hogeveen's dication **2** [5], including x‐ray crystal structures, published in 2013 and 2017 by the groups of Meyer and Krossing [6], and Seppelt [7].

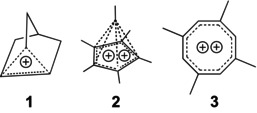

We have now taken a fresh look at the Hückel‐aromatic 1,3,5,7‐tetramethylcycloocta‐tetraene dication **3**, and its rearrangement products.

### 1.1 Computational Methodology

We optimized the geometries of all dications using the M06‐2X/cc‐pVTZ [8, 9], ωB97X‐D/def2‐TZVP [10, 11], and MP2/cc‐pVTZ [9, 12] model chemistries, followed by calculation of force constants and vibrational frequencies. Minima and transition structures are verified as such by zero or exactly one imaginary frequency. Intrinsic reaction coordinate computations [13] for all transition structures at the ωB97X‐D/def2‐TZVP level ascertain the desired nature of the reaction paths. Finally, we computed single point energies on all geometries using DLPNO‐CCSD(T)/cc‐pVQZ [14]. The lowest energies at this level were obtained for the MP2/cc‐pVTZ geometries. The final energies reported in the text are at DLPNO‐CCSD(T)/cc‐pVQZ//MP2/cc‐pVTZ+ZPE (ZPE = Zero‐Point Vibrational Energy). The results are summarized in Tables 1 and 2. We used the Gaussian 16 [15] and ORCA 6.0.0 [16] programs for the quantum chemistry, GaussView 6.0 [17] and CYLview 20 [18] for visualizations. All computational results are included in the Supporting Information.

**TABLE 1:** Relative energies (in kcal mol^−1^) of single points at DLPNO‐CCSD(T)/cc‐pVQZ on geometries obtained at MP2/cc‐pVTZ, ωB97X‐D/def2‐TZVP, and M06‐2X/cc‐pVTZ.

Dication/level	MP2	ωB97X‐D	M06‐2X
3	0.0	0.4	0.3
TS1	1.3	0.0	3.4
7	0.0	1.1	1.2
TS2	0.0	0.3	0.2
5	0.0	0.1	0.1
TS3	0.0	1.2	1.1
6	0.0	0.2	0.1

**TABLE 2:** Energies (in Hartree) and relative energies (in kcal mol^−1^); (a) MP2/cc‐pVTZ+ZPE; (b) ZPE; (c) DLPNO‐CCSD(T)/cc‐pVQZ//MP2/cc‐pVTZ; (d) Sum of (b) and (c).

Dication/level	(a)	(b)	(c)	(d)	*E* rel
3	−464.86885	0.24497	−465.40398	−465.15901	40.9
TS1	−464.83500	0.23960	−465.36569	−465.12609	61.6
7	−464.84944	0.24278	−465.37916	−465.13638	55.1
TS2	−464.84254	0.24238	−465.37293	−465.13055	58.8
5	−464.87007	0.24551	−465.41501	−465.16949	34.4
TS3	−464.84219	0.24229	−465.37401	−465.13173	58.1
6	−464.92186	0.24444	−465.46870	−465.22426	0.0

## 2 Results and Discussion

Olah et al. prepared **3** in 1976 via two‐electron oxidation of 1,3,5,7‐tetramethylcyclooctatetraene **4** with antimony pentafluoride in sulfuryl chloride–fluoride at −78°C [19, 20]. Three signals at 182.7 (*C*), 170.0 (*C*H), and 33.5 (*C*H_3_) ppm are observed in the ^13^C NMR spectrum and two signals at 3.60 (C*H*_3_) and 10.13 (C*H*) ppm in the ^1^H NMR spectrum. Dication **3** rearranges in solution at −20°C to the *cis*‐1,3,5,7‐tetramethyl‐bicyclo[3.3.0]octadienyl dication **5**, characterized by its ^13^C and ^1^H NMR spectrum; it shows signals at 230.6 (*C*H), 164.5 (*C*), 88.3 (*C*), 20.8 (*C*H_3_), 14.6 (*C*H_3_) ppm in the ^13^C NMR, and 11.33 (C*H*), 3.40 (C*H*_3_), 3.02 (C*H*_3_) ppm in the ^1^H NMR spectrum [19].

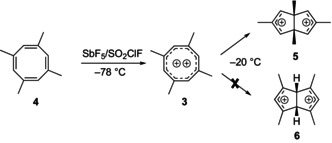

**FIGURE 1:**
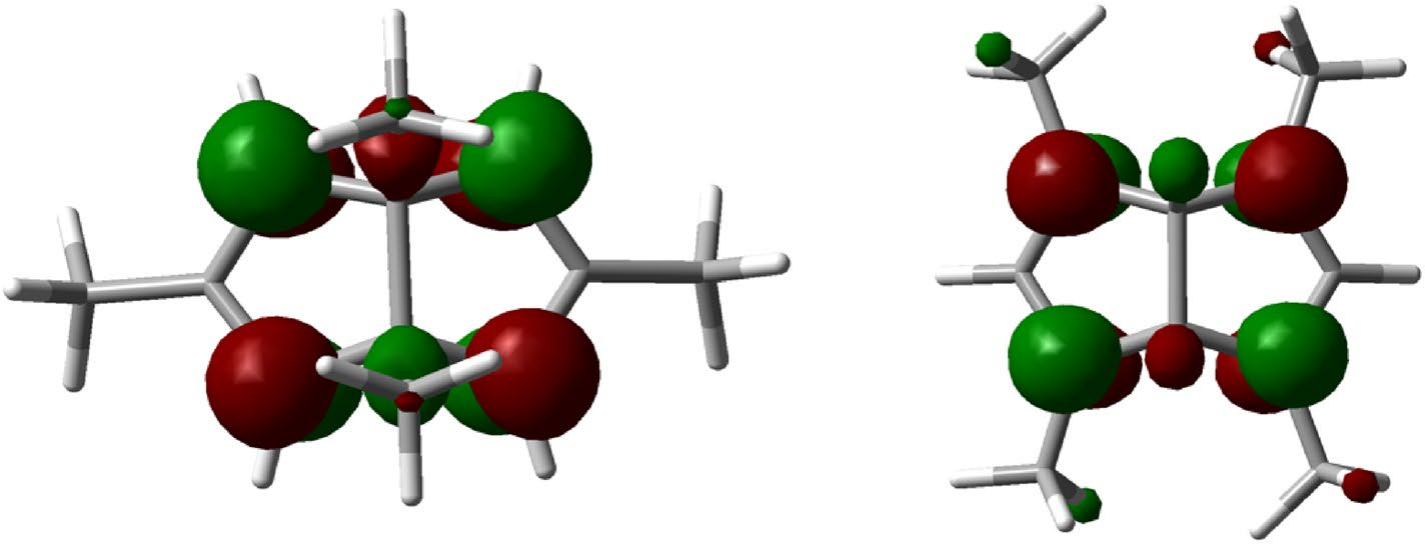
LUMOs of dications **5** (left) and **6**.

Although **5** is lower in energy than **3**, it is not the expected thermodynamically most stable rearrangement product; the two allylic cation moieties would be much more stable in **6** with the methyl groups in the 1,3‐positions, where the LUMO‐coefficients are large (Figure 1).

This unexpected outcome was frequently cited and included in at least one text on Advanced Organic Chemistry [21]—without any commentary. Our computations predict a substantial energy difference of 34.4 kcal mol^−1^ favoring **6** over **5**. Nevertheless, the reported spectroscopic data are unequivocal: Five signals are expected (and observed) in the ^13^C NMR spectrum of the rearrangement product **5**, while **6** would give only four signals.

The authors write: “The conversion [of **3** to **5**] via a concerted mechanistic process is inconsistent with orbital symmetry arguments, but reflects the thermodynamic stability of *cis*‐dihydropentalene derivatives relative to the trans isomers and the importance of maintenance of optimal C_pπ_–C_pπ_ overlap in the allylic fragments of the dications” [20].

Olah et al. were aware of the symmetry‐forbidden direct ring closure and the stability of *cis*‐ over *trans*‐bicyclo[3.3.0]octanes and their geometries, but they left the unexpected substitution pattern uncommented.

The original papers offer no mechanistic explanation of how **3** would rearrange to **5**. Using computations, we have identified a two‐step process from **3** to **5** that first involves contraction of the eight‐membered ring to form a “half‐opened” cyclopropyl cation annulated with a tropylium cation—the novel 2,4,6,8‐tetramethylhomotropylium dication **7**. The parent homotropylium monocation [22] is well known—a classic example for homoaromaticity [23] in carbocations, and the 1,3,5,7‐tetramethyl homotropylium cation **8** forms as a side product during the generation of **3** via protonation of **4** by small amounts of acid (HF) present in the SbF_5_/SO_2_ClF solution [19].

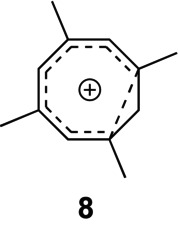

A homoaromatic *dication* such as **7** has never been reported but appears to be a viable structure stabilized by four methyl groups, one directly next to the charge bearing “cyclopropyl” carbon.

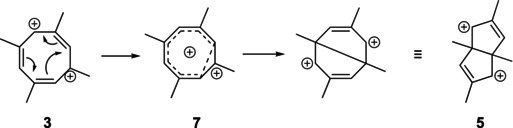

Dication **7** is (homo)aromatic with six π‐electrons but conjoined with a more localized cation with allylic/cyclopropylic character. Half‐opened cyclopropyl cations in ring systems were implied in solvolysis reactions and characterized experimentally [24] and computationally [25]. The barrier for the ring closure is 20.7 kcal mol^−1^ and dication **7** is 14.2 kcal mol^−1^ higher in energy than **3**.

**FIGURE 2:**
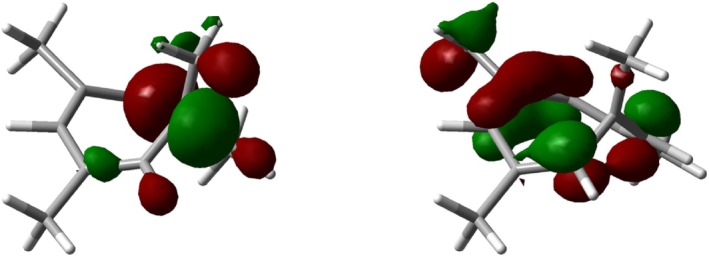
LUMO (left) and HOMO of the transition structure **TS1** connecting **3** and **7**.

The next step is the trans‐annular ring closure to the bicyclo[3.3.0] system, involving the empty orbital at the cyclopropyl moiety and the HOMO of the tropylium system (Figure 2). The computed barrier to reach transition state **TS2** is only 3.7 kcal mol^−1^. We arrive at dication **5** and gain −6.5 kcal mol^−1^ versus the starting dication **3**. Under the experimental conditions reported by Olah et al., the rearrangement now stops.

The isomeric 1,3,5,7‐tetramethylhomotropylium dication **7a** is not a stationary point but collapses to dication **6** upon attempted optimization. An alternative pathway through **7a** is therefore clearly ruled out.

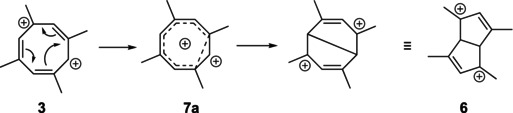

**FIGURE 3:**
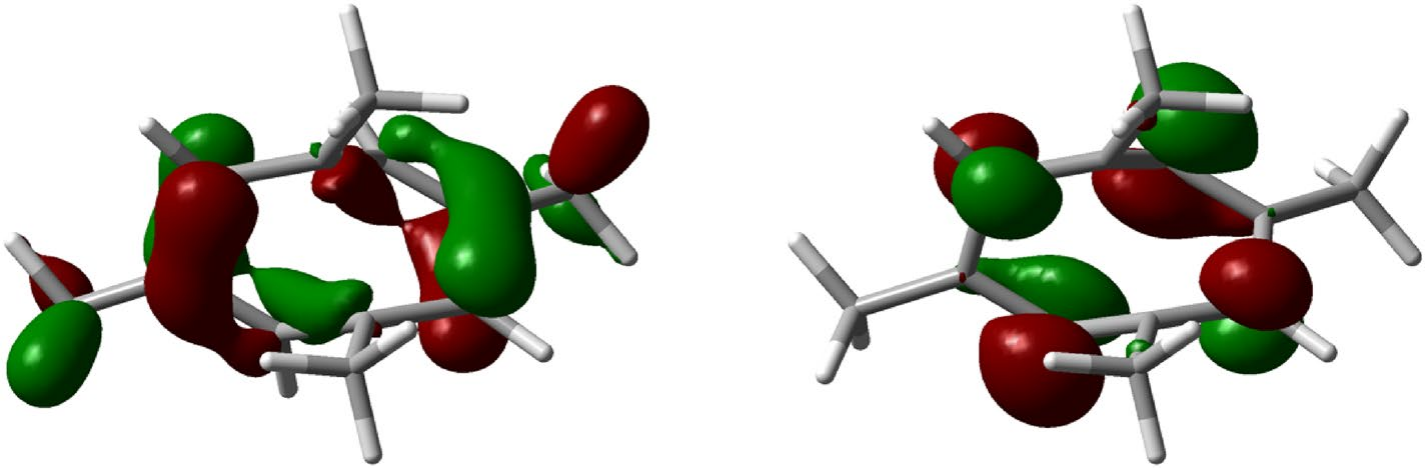
HOMO (left) and LUMO of the transition structure **TS3** between **5** and **6**.

Dications **5** and **6** can interconvert via a _σ_2_s_ + _π_2_a_ + _π_2_a_ Cope rearrangement [26, 27, 28], and we found a transition structure **TS3** between **5** and **6** with a barrier of 23.7 kcal mol^−1^ (Figure 3).

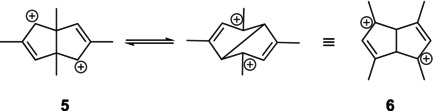

**SCHEME 1:**
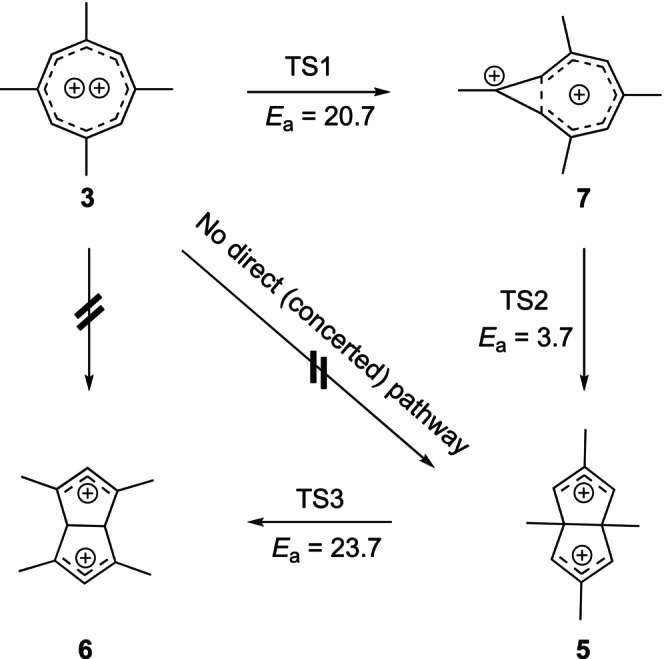
Minima and transition structures in the proposed mechanism from **3** to **6**. Energies of activation (*E*_a_) are in kcal mol^‐1^.

**SCHEME 2:**
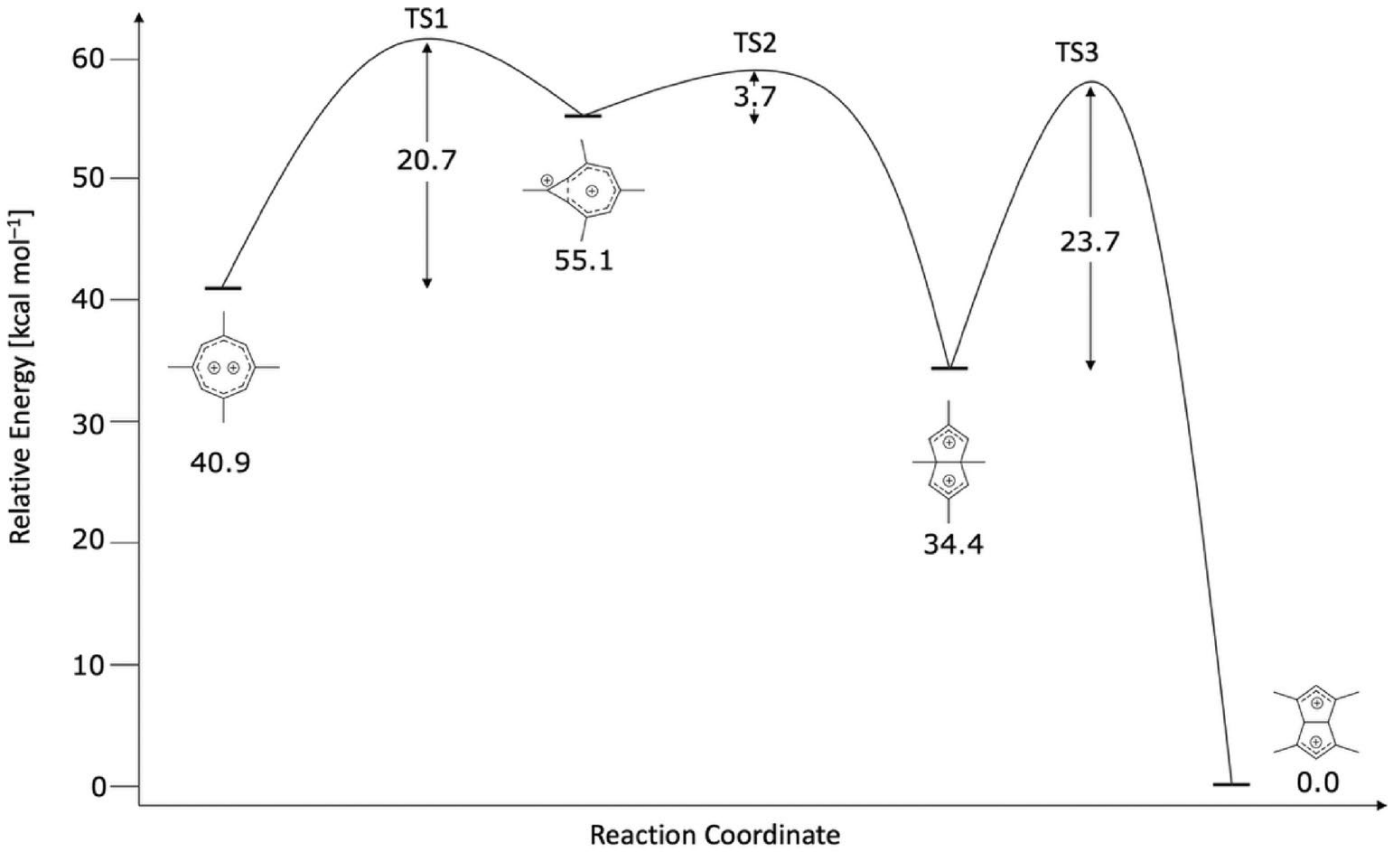
Reaction profile with relative energies at DLPNO‐CCSD(T)/cc‐pVQZ//MP2/cc‐pVTZ+ZPE.

**SCHEME 3:**
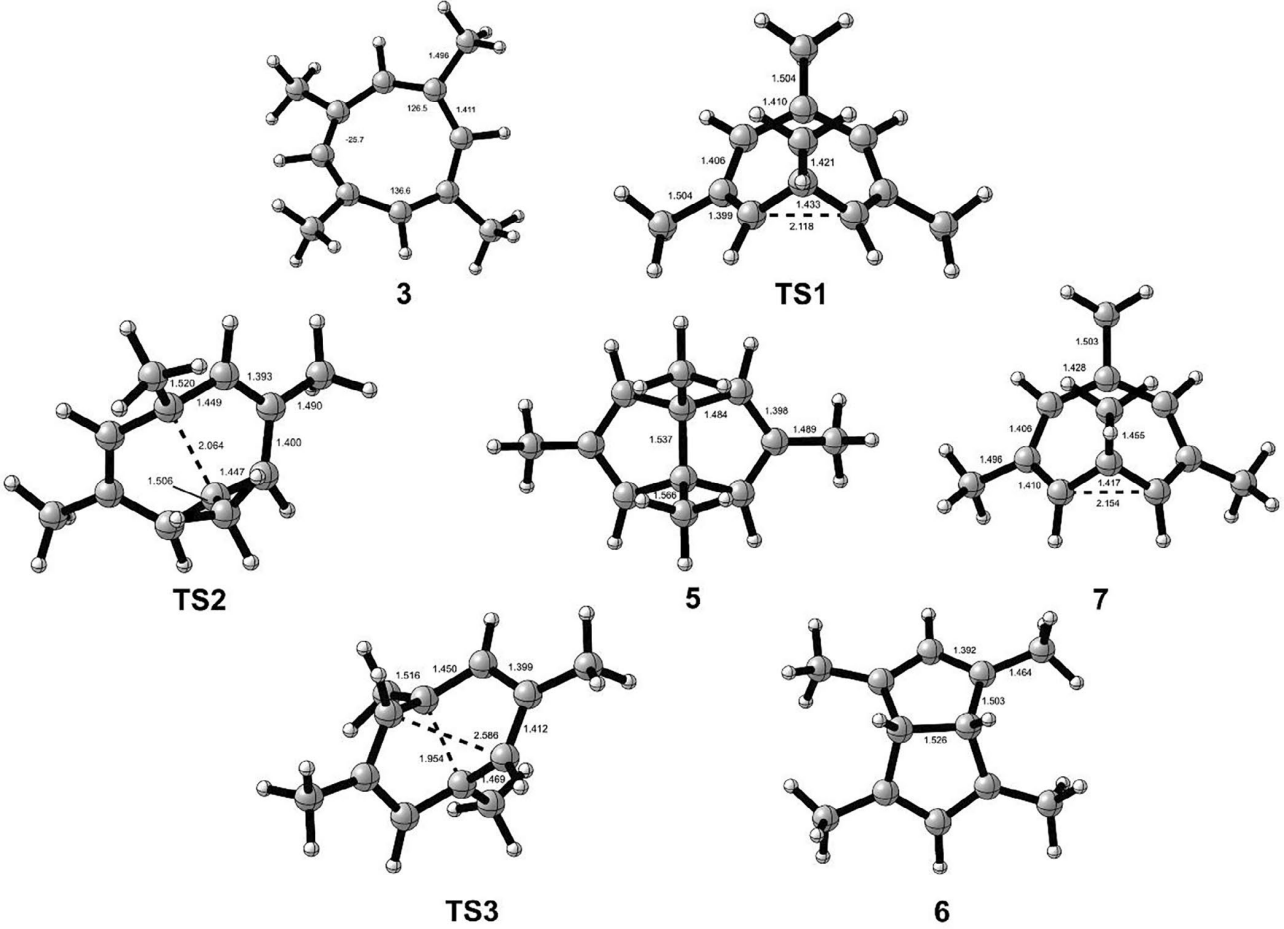
Structures of minima and transition states at MP2/cc‐pVTZ.

This rearrangement is thermodynamically highly favorable but **6** was never observed. If we take the computed relative energies and activation energies at face value, it certainly could have formed; although the barrier is 3.0 kcal mol^−1^ higher than the barrier for the first step in our proposed mechanism. The observed products are kinetically controlled under the reaction conditions. Schemes 1 and 2 summarize the results graphically. The computed structures are shown in Scheme 3.

The 6π‐Hückel‐aromatic parent C_8_H_8_^2+^ **9** dication could not be prepared under stable ion conditions [19], and octamethylcyclooctatetraene **10** is directly oxidized to the octamethyl‐bicyclo[3.3.0]octadienyl dication **11** [19]; here, the substitution pattern cannot be disputed.

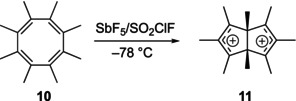

According to basic Hückel theory, cyclooctatetraene dications should be planar. In the case of **3**, a puckered conformation with *D*_2*d*_ symmetry is a minimum lying 4.6 kcal mol^−1^ lower in energy (ωB97X‐D/def2‐TZVP+ZPE) than the planar *C*_4*h*_ symmetric structure that is a second order saddle point. The parent dication **9** is a minimum with *D*_*8h*_ symmetry at MP2/cc‐pVTZ, but it is predicted to be non‐planar (*D*_*2d*_‐symmetry) at ωB97X‐D/def2‐TZVP.

## 3 Conclusions

Olah et al. added cyclooctatetraene dications to the family of experimentally characterized Hückel‐aromatic molecules. They interpreted the observations correctly but left out a seemingly secondary, yet crucial detail: Dication **5** is not the expected most stable rearrangement product; it is dication **6**. Future experimental work to corroborate our computational study is desirable: Will the final rearrangement step take place at temperatures above −20°C or will decomposition ensue?

## Conflicts of Interest

The author declares no conflicts of interest.

## Supporting information


**Data S1:** Supporting Information.

## Data Availability

Data sharing not applicable to this article as no datasets were generated or analyzed during the current study.
