# Supplementary material for: The 2,4,6,8‐Tetramethylhomotropyliumdication
Source: J Comput Chem. 2025 Aug 20;46(23):e70218. doi: 10.1002/jcc.70218 (PMC12494003; doi:10.1002/jcc.70218)

**The 2,4,6,8-Tetramethylhomotropyliumdication**

**Matthias Bremer^1^**

**^1^ Justus-Liebig-Universität, Institut für Organische Chemie, Heinrich-Buff-Ring 17, 35392 Giessen, Germany**

**Supplementary Material**

**Contents**

1. Computations at the wB97X-D/def2-TZVP level of theory

1.1. Data for Dication 3

1.2. Data for Dication 3 in C4h-symmetry

1.3. Data for TS1

1.4. Data for Dication 7

1.5. Data for TS2

1.6. Data for Dication 5

1.7. Data for TS3

1.8. Data for Dication 6

2. IRC-Paths at wB97X-D/def2-TZVP

2.1. TS1

2.2. TS2

2.3. TS3

3. Computations at the M06-2X/cc-pVTZ level of theory

3.1. Data for Dication 3

3.2. Data for TS1

3.3. Data for Dication 7

3.4. Data for TS2

3.5. Data for Dication 5

3.6. Data for TS3

3.7. Data for Dication 6

4. Computations at the MP2/cc-pVTZ level of theory

4.1. Data for Dication 3

4.2. Data for TS1

4.3. Data for Dication 7

4.4. Data for TS2

4.5. Data for Dication 5

4.6. Data for TS3

4.7. Data for Dication 6

5. DLPNO-CCSD(T)/cc-pVQZ single point energies on all geometries

6. Energies (in Hartree) and relative energies (in kcal mol^–1^) at wB97X-D/def2-TZVP//wB97X- D/def2-TZVP

7. Geometries at wB97X-D/def2-TZVP

1. **Computations at the wB97X-D/def2-TZVP level of theory**
   1. **Data for Dication 3**

Data from file me4cot++-w-d2d.log

Molecular Weight = 160.26

28

Extracted Standard Orientation:

C 1.246430 1.246430 -0.000000

H 2.017363 2.017363 -0.000000

C 1.758100 -0.000000 0.391284

C 1.246430 -1.246430 -0.000000

H 2.017363 -2.017363 -0.000000

C 0.000000 1.758100 -0.391284

C -1.246430 1.246430 -0.000000

H -2.017363 2.017363 -0.000000

C -0.000000 -1.758100 -0.391284

C -1.246430 -1.246430 -0.000000

H -2.017363 -2.017363 -0.000000

C -1.758100 -0.000000 0.391284

C 3.121284 -0.000000 1.004782

H 3.279919 -0.889163 1.614393

H 3.279919 0.889163 1.614393

H 3.884442 -0.000000 0.217187

C -0.000000 -3.121284 -1.004782

H -0.000000 -3.884442 -0.217187

H 0.889163 -3.279919 -1.614393

H -0.889163 -3.279919 -1.614393

C -3.121284 -0.000000 1.004782

H -3.279919 -0.889163 1.614393

H -3.884442 -0.000000 0.217187

H -3.279919 0.889163 1.614393

C 0.000000 3.121284 -1.004782

H 0.000000 3.884442 -0.217187

H -0.889163 3.279919 -1.614393

H 0.889163 3.279919 -1.614393

Zero-point correction= 0.243574 (Hartree/Particle)

SCF Done: E(RwB97XD) = -466.176361823 A.U. after 1 cycles

Sum of electronic and zero-point Energies= -465.932788

Sum of electronic and thermal Energies= -465.918975

Sum of electronic and thermal Enthalpies= -465.918030

Sum of electronic and thermal Free Energies= -465.971931

- 1. **Data for Dication 3 in C4h-symmetry**

Data from file me4cot++-c4h-w.log

Molecular Weight = 160.26

28

Extracted Standard Orientation:

C 0.437309 1.756594 0.000000

H 0.703854 2.809456 0.000000

C -0.957786 1.594542 0.000000

C 1.594542 0.957786 0.000000

C 1.756594 -0.437309 0.000000

H 2.809456 -0.703854 0.000000

C 0.957786 -1.594542 0.000000

C -0.437309 -1.756594 0.000000

H -0.703854 -2.809456 0.000000

C -1.756594 0.437309 0.000000

H -2.809456 0.703854 0.000000

C -1.594542 -0.957786 0.000000

C 2.865787 1.756594 0.000000

H 2.892843 2.410260 0.877199

H 2.892843 2.410260 -0.877199

H 3.759170 1.139027 0.000000

C 1.756594 -2.865787 0.000000

H 2.410260 -2.892843 0.877199

H 2.410260 -2.892843 -0.877199

H 1.139027 -3.759170 0.000000

C -2.865787 -1.756594 0.000000

H -2.892843 -2.410260 0.877199

H -2.892843 -2.410260 -0.877199

H -3.759170 -1.139027 0.000000

C -1.756594 2.865787 0.000000

H -2.410260 2.892843 0.877199

H -2.410260 2.892843 -0.877199

H -1.139027 3.759170 0.000000

Zero-point correction= 0.243878 (Hartree/Particle)

SCF Done: E(RwB97XD) = -466.169356578 A.U. after 1 cycles

Sum of electronic and zero-point Energies= -465.925479

Sum of electronic and thermal Energies= -465.912856

Sum of electronic and thermal Enthalpies= -465.911912

Sum of electronic and thermal Free Energies= -465.964268

Dipole moment (field-independent basis, Debye):

X= -0.0000 Y= 0.0000 Z= 0.0000 Tot= 0.0000

****** 2 imaginary frequencies (negative Signs) ******

Frequencies -- -71.4793 -56.2378

- 1. **Data for TS1**

Data from file ts1-w.log

Molecular Weight = 160.26

28

Extracted Standard Orientation:

C 1.132354 -1.381312 -0.185014

H 1.523801 -2.330250 -0.561036

C 1.708173 -0.194805 -0.532627

C 1.247750 1.048384 -0.016520

H 2.031304 1.804425 0.012769

C -0.000006 -1.505582 0.698132

C -1.132364 -1.381306 -0.185016

H -1.523819 -2.330240 -0.561039

C 0.000006 1.548605 0.372628

C -1.247742 1.048393 -0.016520

H -2.031291 1.804439 0.012768

C -1.708173 -0.194793 -0.532629

C 2.957587 -0.179643 -1.368458

H 3.789225 0.223756 -0.786551

H 2.817824 0.466951 -2.236270

H 3.229520 -1.174440 -1.713035

C 0.000012 2.915285 1.013298

H 0.000046 3.693997 0.246140

H 0.882977 3.057096 1.634694

H -0.882979 3.057128 1.634649

C -2.957589 -0.179624 -1.368458

H -3.789238 0.223721 -0.786529

H -3.229493 -1.174413 -1.713081

H -2.817847 0.467015 -2.236239

C -0.000008 -1.826743 2.087992

H -0.923990 -1.571154 2.608193

H 0.923974 -1.571158 2.608194

H -0.000011 -2.948023 2.035517

Zero-point correction= 0.239938 (Hartree/Particle)

SCF Done: E(RwB97XD) = -466.143916466 A.U. after 17 cycles

Sum of electronic and zero-point Energies= -465.903978

Sum of electronic and thermal Energies= -465.890521

Sum of electronic and thermal Enthalpies= -465.889577

Sum of electronic and thermal Free Energies= -465.943362

Dipole moment (field-independent basis, Debye):

X= -0.0000 Y= -3.5813 Z= 1.9426 Tot= 4.0743

****** 1 imaginary frequencies (negative Signs) ******

Frequencies -- -300.7142

- 1. **Data for Dication 7**

Data from file me4bic510dicat-w.log

Molecular Weight = 160.26

28

Extracted Standard Orientation:

C -0.185649 0.988848 1.300647

H -0.272903 1.775087 2.045475

C -0.454164 -0.270723 1.771678

C -0.185649 -1.425757 0.947872

H -0.775679 -2.327175 1.090457

C 0.098670 1.507412 0.000000

C -0.185649 0.988848 -1.300647

H -0.272903 1.775087 -2.045475

C 0.846825 -1.391904 -0.000000

C -0.185649 -1.425757 -0.947872

H -0.775679 -2.327175 -1.090457

C -0.454164 -0.270723 -1.771678

C -1.062467 -0.507777 3.109739

H -2.026292 -1.013585 2.986913

H -1.228132 0.421541 3.648742

H -0.434300 -1.169155 3.711834

C 2.236998 -1.002219 -0.000000

H 2.760056 -1.980269 -0.000000

H 2.545287 -0.493404 0.912489

H 2.545287 -0.493404 -0.912489

C -1.062467 -0.507777 -3.109739

H -2.026292 -1.013585 -2.986913

H -0.434300 -1.169155 -3.711834

H -1.228132 0.421541 -3.648742

C 0.548014 2.928661 0.000000

H 1.119411 3.176729 -0.893624

H 1.119411 3.176729 0.893624

H -0.342737 3.573391 0.000000

Zero-point correction= 0.242193 (Hartree/Particle)

SCF Done: E(RwB97XD) = -466.155243552 A.U. after 1 cycles

Sum of electronic and zero-point Energies= -465.913051

Sum of electronic and thermal Energies= -465.899644

Sum of electronic and thermal Enthalpies= -465.898700

Sum of electronic and thermal Free Energies= -465.952175

Dipole moment (field-independent basis, Debye):

X= 0.6635 Y= -2.2155 Z= -0.0000 Tot= 2.3127

- 1. **Data for TS2**

Data from file ts2a-w.log

Molecular Weight = 160.26

28

Extracted Standard Orientation:

C -1.304432 0.557005 -0.830428

H -1.910556 1.198053 -1.469188

C -1.863039 -0.596292 -0.300220

C -1.105206 -1.054361 0.767891

H -1.226581 -2.048882 1.195090

C 0.000036 1.087628 -0.525685

C 1.304641 0.557238 -0.830129

H 1.910947 1.198553 -1.468441

C -0.000005 -0.221060 1.174972

C 1.105255 -1.054332 0.767952

H 1.226666 -2.048839 1.195171

C 1.863160 -0.596189 -0.300056

C -3.107076 -1.247929 -0.800976

H -2.857918 -2.207866 -1.260446

H -3.599748 -0.633047 -1.551605

H -3.804952 -1.440795 0.013743

C -0.000055 0.876211 2.192948

H -0.000142 0.360014 3.162019

H -0.899259 1.486894 2.143443

H 0.899188 1.486844 2.143567

C 3.107026 -1.247981 -0.801021

H 2.856322 -2.203511 -1.268848

H 3.801409 -1.449313 0.014564

H 3.604225 -0.629068 -1.545354

C -0.000148 2.601771 -0.485630

H 0.887429 2.987766 0.015095

H -0.887999 2.987594 0.014738

H 0.000025 2.985344 -1.511262

Zero-point correction= 0.242176 (Hartree/Particle)

SCF Done: E(RwB97XD) = -466.141895052 A.U. after 1 cycles

Sum of electronic and zero-point Energies= -465.899720

Sum of electronic and thermal Energies= -465.886708

Sum of electronic and thermal Enthalpies= -465.885764

Sum of electronic and thermal Free Energies= -465.940439

Dipole moment (field-independent basis, Debye):

X= -0.0007 Y= -0.5143 Z= 0.6955 Tot= 0.8650

****** 1 imaginary frequencies (negative Signs) ******

Frequencies -- -353.7226

- 1. **Data for Dication 5**

Data from file me4bic330++2a-w.log

Molecular Weight = 160.26

28

Extracted Standard Orientation:

C 1.107228 1.249182 -0.283148

C -1.107386 1.249170 -0.282966

C -0.000119 2.013780 -0.622562

C 1.107386 -1.249170 -0.282966

C -1.107228 -1.249182 -0.283148

C 0.000119 -2.013780 -0.622562

C -0.770261 -0.000041 0.450283

C 0.770261 0.000041 0.450283

H 2.132931 1.528027 -0.512466

H -2.133131 1.528013 -0.512090

H 2.133131 -1.528013 -0.512090

H -2.132931 -1.528027 -0.512466

C 0.000119 -3.348796 -1.283803

H 0.887066 -3.481887 -1.901562

H -0.885347 -3.480627 -1.903971

H -0.001503 -4.134423 -0.524695

C -0.000119 3.348796 -1.283803

H 0.885347 3.480627 -1.903971

H 0.001503 4.134423 -0.524695

H -0.887066 3.481887 -1.901562

C 1.498731 0.000269 1.833508

H 2.578069 0.000424 1.688720

H 1.217437 -0.886914 2.399168

H 1.217151 0.887447 2.399033

C -1.498731 -0.000269 1.833508

H -1.217437 0.886914 2.399168

H -1.217151 -0.887447 2.399033

H -2.578069 -0.000424 1.688720

Zero-point correction= 0.245452 (Hartree/Particle)

SCF Done: E(RwB97XD) = -466.185693803 A.U. after 1 cycles

Sum of electronic and zero-point Energies= -465.940242

Sum of electronic and thermal Energies= -465.927292

Sum of electronic and thermal Enthalpies= -465.926348

Sum of electronic and thermal Free Energies= -465.978933

Dipole moment (field-independent basis, Debye):

X= -0.0000 Y= 0.0000 Z= -0.6158 Tot= 0.6158

- 1. **Data for TS3**

Data from file ts3a-w.log

Molecular Weight = 160.26

28

Extracted Standard Orientation:

C 1.902386 -0.597282 0.071693

C 0.032234 0.470991 1.028937

C 1.445782 0.186309 1.113132

H 2.075199 0.575849 1.907603

C -0.032234 0.470990 -1.028937

C -1.902387 -0.597282 -0.071692

C -1.445782 0.186309 -1.113132

H -2.075199 0.575849 -1.907603

C -0.872779 -0.667494 0.894658

H -0.771272 -1.513588 1.573818

C 0.872779 -0.667495 -0.894658

H 0.771272 -1.513590 -1.573816

C 0.500068 1.764676 -1.586713

H -0.138656 2.601351 -1.307596

H 0.493233 1.685245 -2.679340

H 1.525634 1.957212 -1.278313

C -3.210089 -1.300043 0.018582

H -3.064325 -2.318206 0.387079

H -3.714051 -1.338943 -0.943862

H -3.858333 -0.791871 0.736963

C 3.210088 -1.300043 -0.018581

H 3.714042 -1.338962 0.943867

H 3.858340 -0.791856 -0.736945

H 3.064329 -2.318198 -0.387101

C -0.500067 1.764678 1.586711

H -0.493234 1.685247 2.679338

H -1.525633 1.957214 1.278311

H 0.138658 2.601352 1.307595

Zero-point correction= 0.242120 (Hartree/Particle)

SCF Done: E(RwB97XD) = -466.142408841 A.U. after 1 cycles

Sum of electronic and zero-point Energies= -465.900289

Sum of electronic and thermal Energies= -465.887471

Sum of electronic and thermal Enthalpies= -465.886527

Sum of electronic and thermal Free Energies= -465.938395

Dipole moment (field-independent basis, Debye):

X= 0.0000 Y= -0.4445 Z= 0.0000 Tot= 0.4445

****** 1 imaginary frequencies (negative Signs) ******

Frequencies -- -337.9060

- 1. **Data for Dication 6**

Data from file me4bic330++-w.log

Molecular Weight = 160.26

28

Extracted Standard Orientation:

C 1.727792 0.077038 0.051223

C 0.122932 1.699952 -0.009804

C 1.420553 1.366110 -0.349478

H 2.112012 2.032741 -0.847841

C -0.122932 -1.699952 -0.009804

C -1.727792 -0.077038 0.051223

C -1.420553 -1.366110 -0.349478

H -2.112012 -2.032741 -0.847841

C -0.529528 0.548069 0.712948

H -0.808434 0.887488 1.720341

C 0.529528 -0.548069 0.712948

H 0.808434 -0.887488 1.720341

C 0.529528 -2.982220 -0.282110

H -0.191632 -3.769985 -0.492923

H 1.198447 -3.282779 0.526047

H 1.151499 -2.865092 -1.182515

C -3.038086 0.561220 -0.058363

H -3.560531 0.365519 0.892788

H -3.646731 0.124900 -0.848995

H -2.986241 1.644877 -0.153399

C 3.038086 -0.561220 -0.058363

H 3.646731 -0.124900 -0.848995

H 2.986241 -1.644877 -0.153399

H 3.560531 -0.365519 0.892788

C -0.529528 2.982220 -0.282110

H -1.198447 3.282779 0.526047

H -1.151499 2.865092 -1.182515

H 0.191632 3.769985 -0.492923

Zero-point correction= 0.244641 (Hartree/Particle)

SCF Done: E(RwB97XD) = -466.248363584 A.U. after 1 cycles

Sum of electronic and zero-point Energies= -466.003723

Sum of electronic and thermal Energies= -465.990981

Sum of electronic and thermal Enthalpies= -465.990036

Sum of electronic and thermal Free Energies= -466.041909

Dipole moment (field-independent basis, Debye):

X= 0.0000 Y= 0.0000 Z= 0.3455 Tot= 0.3455

1. **IRC-Paths at wB97X-D/def2-TZVP**
   1. **TS1**


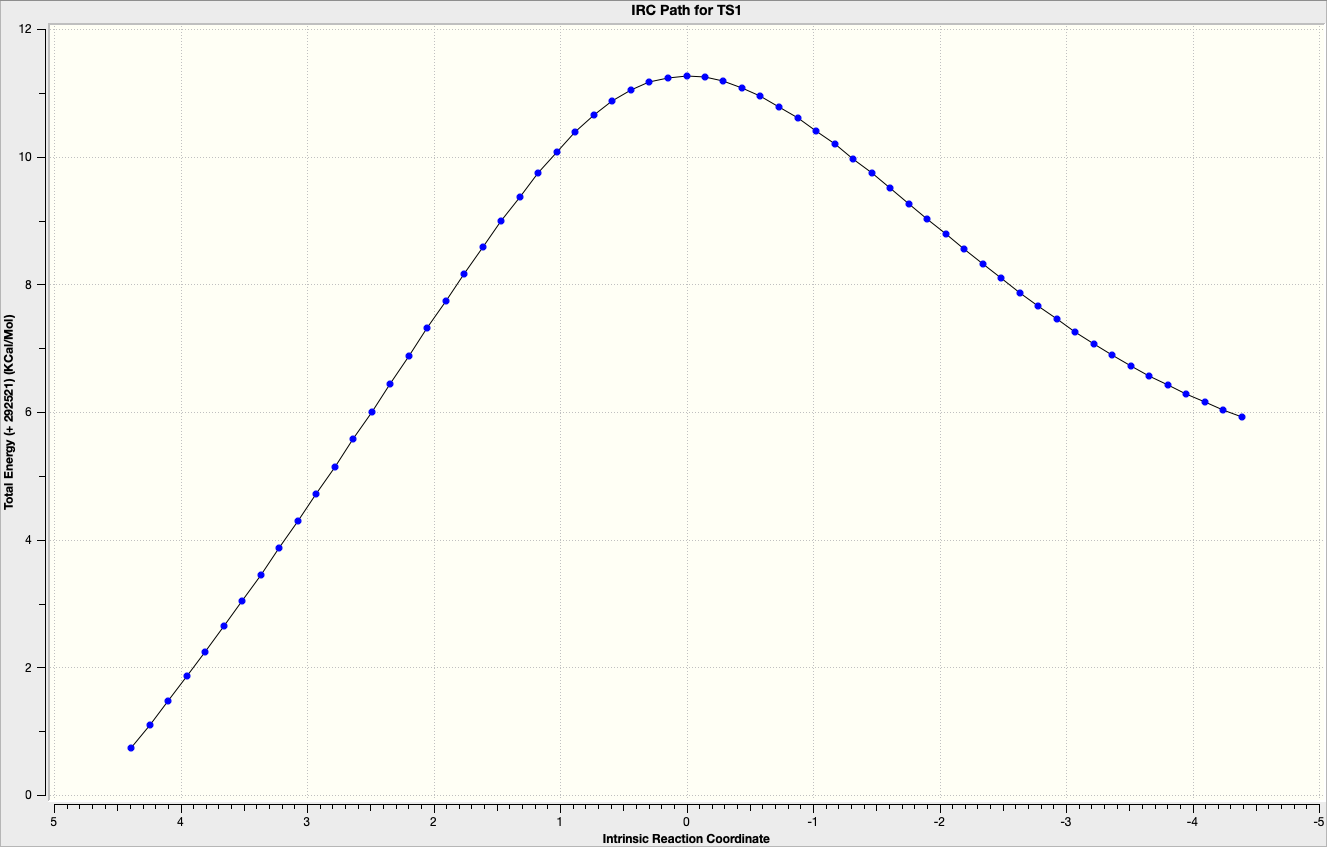


- 1. **TS2**

**
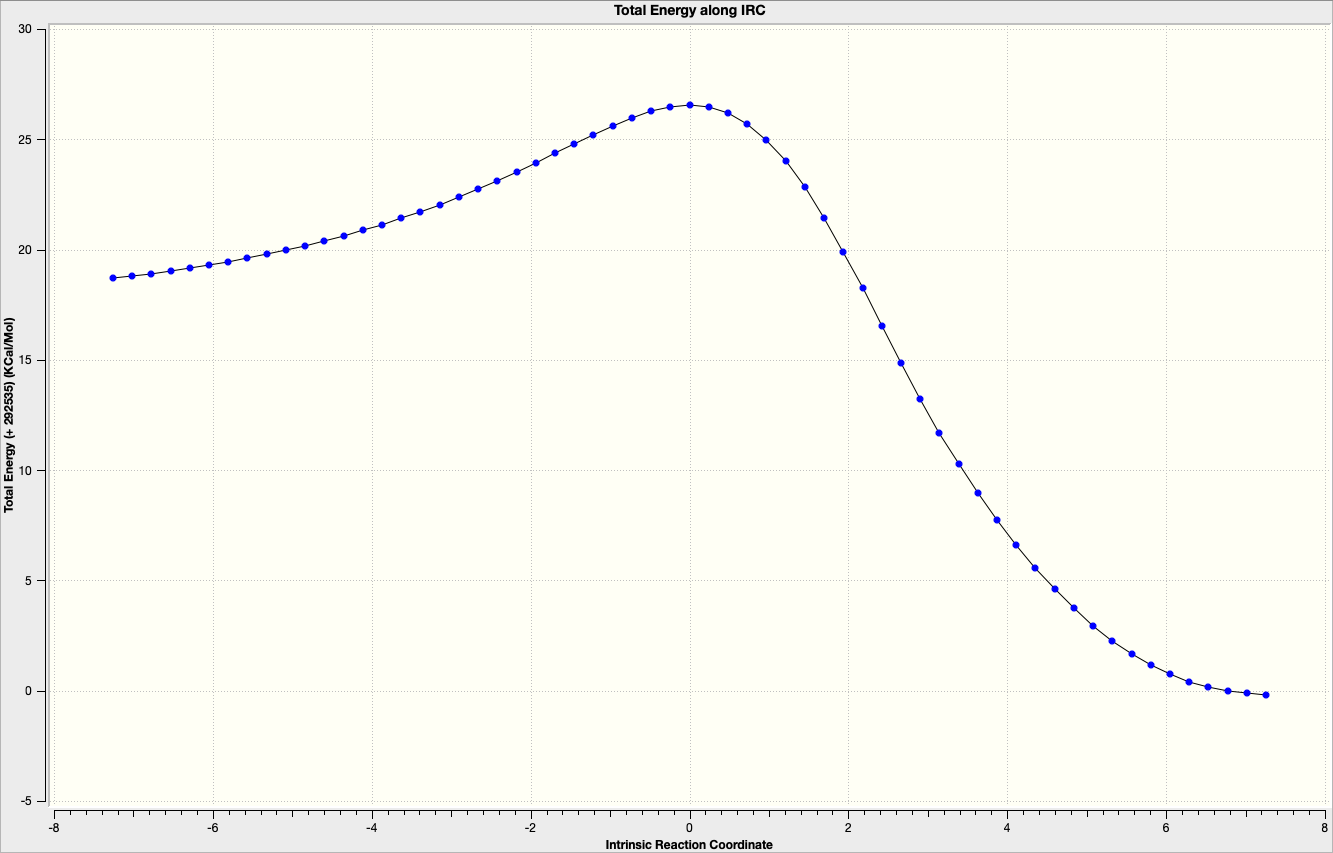
**

- 1. **TS3**


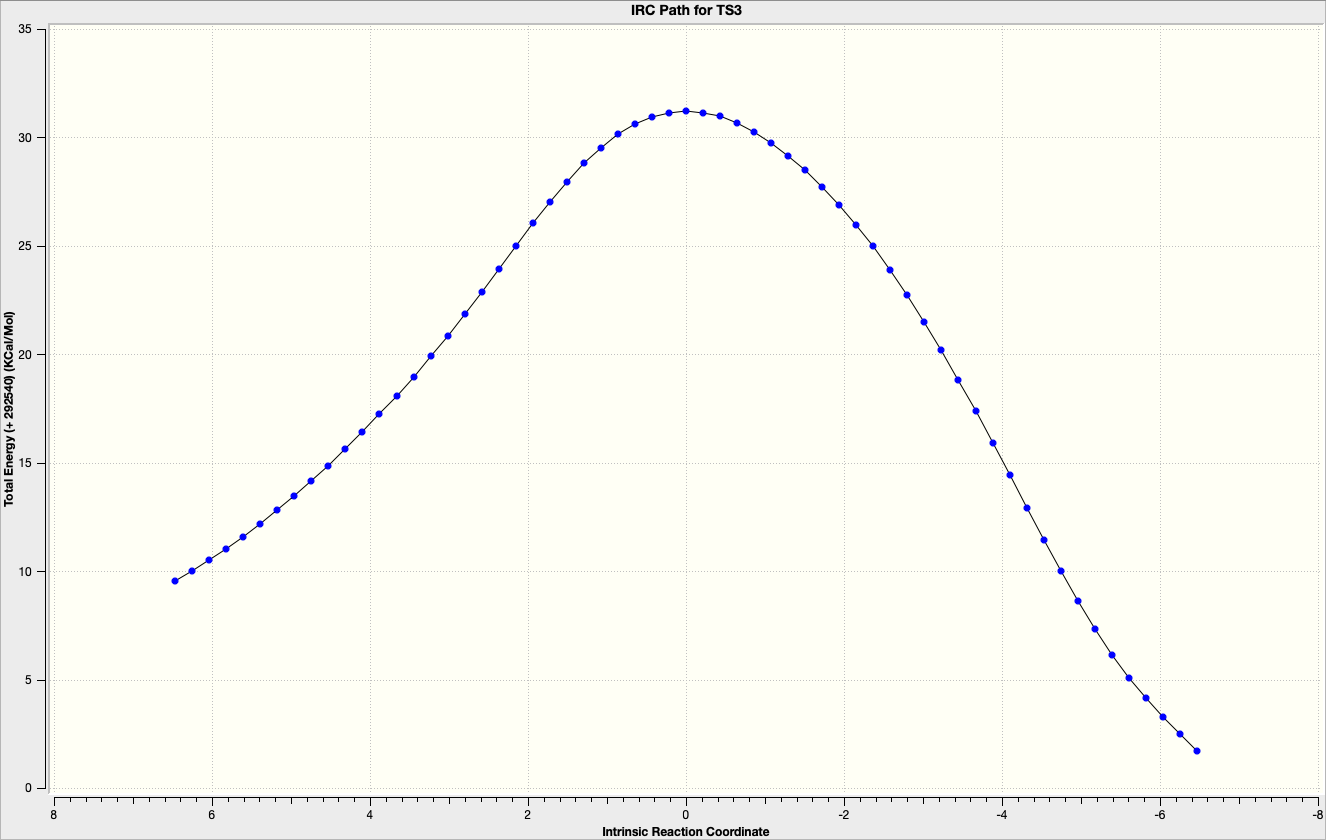


1. **Computations at the M06-2X/cc-pVTZ level of theory**
   1. **Data for Dication 3**

Data from file COT++.log

Molecular Weight = 160.26

28

Extracted Standard Orientation:

C 1.247277 1.247277 0.000000

H 2.018645 2.018645 0.000000

C 1.758471 0.000000 0.393964

C 1.247277 -1.247277 0.000000

H 2.018645 -2.018645 0.000000

C 0.000000 1.758471 -0.393964

C -1.247277 1.247277 0.000000

H -2.018645 2.018645 0.000000

C 0.000000 -1.758471 -0.393964

C -1.247277 -1.247277 0.000000

H -2.018645 -2.018645 0.000000

C -1.758471 0.000000 0.393964

C 3.123652 0.000000 1.003206

H 3.282799 -0.890116 1.609445

H 3.282799 0.890116 1.609445

H 3.881377 0.000000 0.210593

C 0.000000 -3.123652 -1.003206

H 0.000000 -3.881377 -0.210593

H 0.890116 -3.282799 -1.609445

H -0.890116 -3.282799 -1.609445

C -3.123652 0.000000 1.003206

H -3.282799 -0.890116 1.609445

H -3.881377 0.000000 0.210593

H -3.282799 0.890116 1.609445

C 0.000000 3.123652 -1.003206

H 0.000000 3.881377 -0.210593

H -0.890116 3.282799 -1.609445

H 0.890116 3.282799 -1.609445

Zero-point correction= 0.241500 (Hartree/Particle)

SCF Done: E(RM062X) = -466.094536844 A.U. after 1 cycles

Sum of electronic and zero-point Energies= -465.853037

Sum of electronic and thermal Energies= -465.842090

Sum of electronic and thermal Enthalpies= -465.841146

Sum of electronic and thermal Free Energies= -465.888430

- 1. **Data for TS1**

Data from file TS1.log

Molecular Weight = 160.26

28

Extracted Standard Orientation:

C -1.270256 -0.921948 -0.349791

H -2.126369 -1.419396 -0.816148

C -0.015423 -1.618146 -0.494897

C 1.183376 -1.248574 0.066838

H 1.920471 -2.045397 0.102192

C -1.713815 -0.005540 0.632934

C -1.276307 0.913590 -0.349970

H -2.135593 1.405387 -0.816476

C 1.690468 0.005508 0.462631

C 1.175131 1.256247 0.066923

H 1.906956 2.057906 0.102328

C -0.026026 1.617960 -0.494941

C -0.132964 -2.926402 -1.214837

H 0.835677 -3.401134 -1.334532

H -0.570504 -2.755146 -2.202440

H -0.799457 -3.608999 -0.682900

C 3.041887 0.009899 1.094404

H 3.804308 0.011493 0.306239

H 3.201420 -0.881445 1.697642

H 3.196282 0.902951 1.696421

C -0.151992 2.925396 -1.214943

H 0.813533 3.406516 -1.334326

H -0.823162 3.603593 -0.683262

H -0.588052 2.751205 -2.202690

C -2.584680 -0.008269 1.763590

H -3.158618 0.910697 1.880322

H -1.840948 -0.005491 2.599222

H -3.152346 -0.931061 1.880768

Zero-point correction= 0.239839 (Hartree/Particle)

SCF Done: E(RM062X) = -466.063086893 A.U. after 1 cycles

Sum of electronic and zero-point Energies= -465.823248

Sum of electronic and thermal Energies= -465.809827

Sum of electronic and thermal Enthalpies= -465.808883

Sum of electronic and thermal Free Energies= -465.863246

Dipole moment (field-independent basis, Debye):

X= -3.9090 Y= -0.0125 Z= 1.4633 Tot= 4.1739

****** 1 imaginary frequencies (negative Signs) ******

Frequencies -- -383.2408

- 1. **Data for Dication 7**

Data from file me4bic510dicat.log

Molecular Weight = 160.26

28

Extracted Standard Orientation:

C -0.185885 0.990354 1.302807

H -0.273817 1.772574 2.051413

C -0.450261 -0.271921 1.766556

C -0.185885 -1.428033 0.932043

H -0.772974 -2.330273 1.079942

C 0.088123 1.513594 0.000000

C -0.185885 0.990354 -1.302807

H -0.273817 1.772574 -2.051413

C 0.861989 -1.395809 0.000000

C -0.185885 -1.428033 -0.932043

H -0.772974 -2.330273 -1.079942

C -0.450261 -0.271921 -1.766556

C -1.047921 -0.521445 3.106982

H -2.007698 -1.034038 2.983667

H -1.215561 0.404292 3.649237

H -0.412144 -1.183684 3.699117

C 2.247041 -0.986484 0.000000

H 2.779353 -1.960558 0.000000

H 2.547093 -0.475989 0.913493

H 2.547093 -0.475989 -0.913493

C -1.047921 -0.521445 -3.106982

H -2.007698 -1.034038 -2.983667

H -0.412144 -1.183684 -3.699117

H -1.215561 0.404292 -3.649237

C 0.504116 2.944260 0.000000

H 1.064729 3.206364 -0.895063

H 1.064729 3.206364 0.895063

H -0.406811 3.561244 0.000000

Zero-point correction= 0.241837 (Hartree/Particle)

SCF Done: E(RM062X) = -466.076931282 A.U. after 1 cycles

Sum of electronic and zero-point Energies= -465.835094

Sum of electronic and thermal Energies= -465.821890

Sum of electronic and thermal Enthalpies= -465.820946

Sum of electronic and thermal Free Energies= -465.873477

Dipole moment (field-independent basis, Debye):

X= 0.6970 Y= -2.1956 Z= 0.0000 Tot= 2.3036

- 1. **Data for TS2**

Data from file TS2.log

Molecular Weight = 160.26

28

Extracted Standard Orientation:

C -1.302928 0.537380 -0.844932

H -1.897198 1.167673 -1.505721

C -1.868201 -0.605054 -0.298059

C -1.103404 -1.048262 0.776002

H -1.218731 -2.038207 1.215212

C 0.001327 1.070980 -0.528088

C 1.311687 0.548030 -0.832570

H 1.913949 1.190059 -1.474195

C 0.000042 -0.203304 1.166140

C 1.105011 -1.047474 0.777257

H 1.221654 -2.037046 1.216911

C 1.872807 -0.600495 -0.292302

C -3.123552 -1.253361 -0.777661

H -2.948961 -2.315384 -0.955509

H -3.474282 -0.799020 -1.700466

H -3.906033 -1.169395 -0.022599

C -0.001046 0.920924 2.162178

H -0.006462 0.427877 3.142301

H -0.898403 1.531088 2.089379

H 0.900355 1.525717 2.095961

C 3.121468 -1.255201 -0.779917

H 2.863880 -2.159149 -1.336665

H 3.764049 -1.543513 0.049664

H 3.671901 -0.595257 -1.446435

C -0.006870 2.587025 -0.499189

H 0.873250 2.979819 0.007992

H -0.904611 2.972938 -0.018626

H 0.007602 2.954663 -1.530355

Zero-point correction= 0.241774 (Hartree/Particle)

SCF Done: E(RM062X) = -466.062491428 A.U. after 15 cycles

Sum of electronic and zero-point Energies= -465.820717

Sum of electronic and thermal Energies= -465.807806

Sum of electronic and thermal Enthalpies= -465.806862

Sum of electronic and thermal Free Energies= -465.859335

Dipole moment (field-independent basis, Debye):

X= -0.0365 Y= -0.4676 Z= 0.6586 Tot= 0.8085

****** 1 imaginary frequencies (negative Signs) ******

Frequencies -- -284.8464

- 1. **Data for Dication 5**

Data from file bic330b.log

Molecular Weight = 160.26

28

Extracted Standard Orientation:

C -1.134220 1.230493 -0.266211

C -1.355313 -0.974617 -0.272964

C -2.008950 0.206515 -0.606954

C 1.355313 0.974617 -0.272964

C 1.134220 -1.230493 -0.266211

C 2.008950 -0.206515 -0.606954

C -0.078085 -0.764560 0.459129

C 0.078085 0.764560 0.459129

H -1.310688 2.280641 -0.486608

H -1.735676 -1.967524 -0.502095

H 1.735676 1.967524 -0.502095

H 1.310688 -2.280641 -0.486608

C 3.339451 -0.338967 -1.267864

H 3.481009 0.440654 -2.014233

H 3.443950 -1.310157 -1.745874

H 4.130982 -0.238813 -0.523432

C -3.339451 0.338967 -1.267864

H -3.443950 1.310157 -1.745874

H -4.130982 0.238813 -0.523432

H -3.481009 -0.440654 -2.014233

C 0.158586 1.483707 1.848394

H 0.266654 2.556900 1.704849

H 1.015857 1.108179 2.404418

H -0.751582 1.287231 2.412322

C -0.158586 -1.483707 1.848394

H -1.015857 -1.108179 2.404418

H 0.751582 -1.287231 2.412322

H -0.266654 -2.556900 1.704849

Zero-point correction= 0.245489 (Hartree/Particle)

SCF Done: E(RM062X) = -466.102529970 A.U. after 1 cycles

Sum of electronic and zero-point Energies= -465.857041

Sum of electronic and thermal Energies= -465.844354

Sum of electronic and thermal Enthalpies= -465.843410

Sum of electronic and thermal Free Energies= -465.894028

Dipole moment (field-independent basis, Debye):

X= 0.0000 Y= 0.0000 Z= -0.4407 Tot= 0.4407

- 1. **Data for TS3**

Data from file TS3.log

Molecular Weight = 160.26

28

Extracted Standard Orientation:

C -1.914570 -0.598057 0.067039

C -0.886004 -0.666706 -0.902263

H -0.789213 -1.509863 -1.586258

C -1.447247 0.172253 1.115414

H -2.069454 0.550376 1.921539

C 0.032048 0.463631 -1.022714

C 1.447251 0.173334 -1.115170

H 2.069507 0.552278 -1.920876

C 1.914519 -0.598088 -0.067594

C 0.885900 -0.667758 0.901583

H 0.788973 -1.511663 1.584627

C -0.032027 0.462534 1.023145

C -0.491084 1.772813 -1.559903

H -1.511655 1.967252 -1.238432

H -0.492506 1.707156 -2.653011

H 0.160131 2.596186 -1.272572

C -3.229119 -1.292727 -0.023168

H -3.897262 -0.733678 -0.682201

H -3.697537 -1.390855 0.951657

H -3.100442 -2.282790 -0.463683

C 3.229081 -1.292805 0.022045

H 3.897299 -0.733965 0.681168

H 3.697333 -1.390506 -0.952909

H 3.100522 -2.283040 0.462193

C 0.491218 1.771210 1.561467

H -0.159885 2.594881 1.274750

H 1.511837 1.965771 1.240211

H 0.492554 1.704655 2.654515

Zero-point correction= 0.241558 (Hartree/Particle)

SCF Done: E(RM062X) = -466.061657410 A.U. after 1 cycles

Sum of electronic and zero-point Energies= -465.820100

Sum of electronic and thermal Energies= -465.807420

Sum of electronic and thermal Enthalpies= -465.806476

Sum of electronic and thermal Free Energies= -465.857456

Dipole moment (field-independent basis, Debye):

X= -0.0001 Y= -0.4009 Z= -0.0004 Tot= 0.4009

****** 1 imaginary frequencies (negative Signs) ******

Frequencies -- -322.5851

- 1. **Data for Dication 6**

Data from file bic330a.log

Molecular Weight = 160.26

28

Extracted Standard Orientation:

C 1.724261 0.071955 0.048090

C 0.122860 1.698942 -0.004489

C 1.418502 1.361858 -0.356297

H 2.106917 2.024381 -0.863609

C -0.122860 -1.698942 -0.004489

C -1.724261 -0.071955 0.048090

C -1.418502 -1.361858 -0.356297

H -2.106917 -2.024381 -0.863609

C -0.529011 0.548787 0.723787

H -0.815791 0.891236 1.727843

C 0.529011 -0.548787 0.723787

H 0.815791 -0.891236 1.727843

C 0.529011 -2.983452 -0.273353

H -0.196798 -3.771533 -0.463074

H 1.213837 -3.273333 0.523955

H 1.129471 -2.873128 -1.189806

C -3.029889 0.576231 -0.069801

H -3.536745 0.429882 0.898700

H -3.653363 0.117335 -0.833728

H -2.962167 1.654414 -0.207912

C 3.029889 -0.576231 -0.069801

H 3.653363 -0.117335 -0.833728

H 2.962167 -1.654414 -0.207912

H 3.536745 -0.429882 0.898700

C -0.529011 2.983452 -0.273353

H -1.213837 3.273333 0.523955

H -1.129471 2.873128 -1.189806

H 0.196798 3.771533 -0.463074

Zero-point correction= 0.243378 (Hartree/Particle)

SCF Done: E(RM062X) = -466.162599328 A.U. after 1 cycles

Sum of electronic and zero-point Energies= -465.919222

Sum of electronic and thermal Energies= -465.906440

Sum of electronic and thermal Enthalpies= -465.905495

Sum of electronic and thermal Free Energies= -465.957180

Dipole moment (field-independent basis, Debye):

X= 0.0000 Y= 0.0000 Z= 0.2914 Tot= 0.2914

1. **Computations at the MP2/cc-pVTZ level of theory**
   1. **Data for Dication 3**

Data from file me4cot++-d2d-mp2.log

Molecular Weight = 160.26

28

Extracted Standard Orientation:

C 1.260320 1.260320 0.000000

H 2.031089 2.031089 -0.000000

C 1.782576 -0.000000 0.361128

C 1.260320 -1.260320 0.000000

H 2.031089 -2.031089 -0.000000

C 0.000000 1.782576 -0.361128

C -1.260320 1.260320 0.000000

H -2.031089 2.031089 -0.000000

C -0.000000 -1.782576 -0.361128

C -1.260320 -1.260320 0.000000

H -2.031089 -2.031089 -0.000000

C -1.782576 -0.000000 0.361128

C 3.170578 -0.000000 0.919598

H 3.349120 -0.889042 1.520734

H 3.349120 0.889042 1.520734

H 3.895704 -0.000000 0.099205

C -0.000000 -3.170578 -0.919598

H -0.000000 -3.895704 -0.099205

H 0.889042 -3.349120 -1.520734

H -0.889042 -3.349120 -1.520734

C -3.170578 -0.000000 0.919598

H -3.349120 -0.889042 1.520734

H -3.895704 -0.000000 0.099205

H -3.349120 0.889042 1.520734

C 0.000000 3.170578 -0.919598

H 0.000000 3.895704 -0.099205

H -0.889042 3.349120 -1.520734

H 0.889042 3.349120 -1.520734

Zero-point correction= 0.244966 (Hartree/Particle)

SCF Done: E(RHF) = -463.181223122 A.U. after 1 cycles

Sum of electronic and zero-point Energies= -464.868845

Sum of electronic and thermal Energies= -464.855029

Sum of electronic and thermal Enthalpies= -464.854084

Sum of electronic and thermal Free Energies= -464.907839

- 1. **Data for TS1**

Data from file cot-htc-ts-mp2-new2.out

Molecular Weight = 160.26

28

Extracted Standard Orientation:

C 1.059367 -1.357337 -0.256299

H 1.474205 -2.280839 -0.669733

C 1.674133 -0.132053 -0.536657

C 1.256975 1.089412 0.020449

H 2.040377 1.844912 0.044107

C 0.000407 -1.558888 0.688311

C -1.058774 -1.357702 -0.256140

H -1.473179 -2.281434 -0.669504

C -0.000375 1.586545 0.422070

C -1.257546 1.088918 0.020508

H -2.041265 1.844087 0.044197

C -1.674169 -0.132752 -0.536534

C 2.946793 -0.152335 -1.337921

H 3.794699 0.034944 -0.677062

H 2.926651 0.636525 -2.087530

H 3.098692 -1.109416 -1.828794

C -0.000660 2.943770 1.069167

H -0.001626 3.718527 0.299375

H 0.885854 3.081258 1.683425

H -0.886540 3.080253 1.684575

C -2.946747 -0.153598 -1.337909

H -3.794560 0.035851 -0.677563

H -3.099470 -1.111505 -1.826894

H -2.925765 0.633747 -2.089107

C 0.000586 -2.060884 2.017991

H -0.924960 -1.892187 2.565511

H 0.926152 -1.891844 2.565378

H 0.000797 -3.161452 1.777400

Zero-point correction= 0.239600 (Hartree/Particle)

SCF Done: E(RHF) = -463.142858628 A.U. after 1 cycles

Sum of electronic and zero-point Energies= -464.834999

Sum of electronic and thermal Energies= -464.821226

Sum of electronic and thermal Enthalpies= -464.820281

Sum of electronic and thermal Free Energies= -464.875449

Dipole moment (field-independent basis, Debye):

X= 0.0012 Y= -4.1787 Z= 1.8594 Tot= 4.5737

****** 1 imaginary frequencies (negative Signs) ******

Frequencies -- -383.1543

- 1. **Data for Dication 7**

Data from file me4bic510dicat-mp2.log

Molecular Weight = 160.26

28

Extracted Standard Orientation:

C -0.361285 0.887365 1.301589

H -0.645246 1.649809 2.024326

C -0.361285 -0.419938 1.817973

C 0.260842 -1.445777 1.077243

H -0.036344 -2.486356 1.206248

C -0.165638 1.440816 0.000000

C -0.361285 0.887365 -1.301589

H -0.645246 1.649809 -2.024326

C 1.097431 -1.061464 -0.000000

C 0.260842 -1.445777 -1.077243

H -0.036344 -2.486356 -1.206248

C -0.361285 -0.419938 -1.817973

C -1.091586 -0.750825 3.081317

H -2.061745 -1.187689 2.830286

H -1.274628 0.147515 3.665815

H -0.540832 -1.470968 3.681721

C 2.338657 -0.301910 -0.000000

H 3.089554 -1.117432 -0.000000

H 2.505972 0.266501 0.911861

H 2.505972 0.266501 -0.911861

C -1.091586 -0.750825 -3.081317

H -2.061745 -1.187689 -2.830286

H -0.540832 -1.470968 -3.681721

H -1.274628 0.147515 -3.665815

C -0.003640 2.934870 0.000000

H 0.523985 3.275526 -0.888445

H 0.523985 3.275526 0.888445

H -0.992970 3.404980 0.000000

Zero-point correction= 0.242775 (Hartree/Particle)

SCF Done: E(RHF) = -463.150067009 A.U. after 1 cycles

Sum of electronic and zero-point Energies= -464.849436

Sum of electronic and thermal Energies= -464.835918

Sum of electronic and thermal Enthalpies= -464.834974

Sum of electronic and thermal Free Energies= -464.889297

Dipole moment (field-independent basis, Debye):

X= 0.9704 Y= -2.0153 Z= -0.0000 Tot= 2.2367

- 1. **Data for TS2**

Data from file ts2a-mp2.log

Molecular Weight = 160.26

28

Extracted Standard Orientation:

C -1.311286 0.509099 -0.863733

H -1.901465 1.123674 -1.542037

C -1.868300 -0.624823 -0.276923

C -1.091383 -1.009304 0.823059

H -1.197669 -1.968578 1.327772

C -0.000003 1.040853 -0.550676

C 1.311259 0.509077 -0.863756

H 1.901431 1.123629 -1.542088

C 0.000007 -0.119630 1.155810

C 1.091373 -1.009324 0.823043

H 1.197644 -1.968606 1.327743

C 1.868275 -0.624852 -0.276948

C -3.111062 -1.309128 -0.732767

H -2.873186 -2.330945 -1.031327

H -3.551894 -0.794312 -1.581524

H -3.839077 -1.358369 0.075221

C 0.000022 1.045244 2.109974

H 0.000033 0.589884 3.106174

H -0.898000 1.650314 2.011698

H 0.898044 1.650309 2.011679

C 3.111060 -1.309125 -0.732778

H 2.873496 -2.331380 -1.030062

H 3.839533 -1.357146 0.074881

H 3.551218 -0.795027 -1.582318

C 0.000011 2.560639 -0.580075

H 0.888815 2.967853 -0.102358

H -0.888770 2.967866 -0.102327

H -0.000005 2.878466 -1.626519

Zero-point correction= 0.242381 (Hartree/Particle)

SCF Done: E(RHF) = -463.139353648 A.U. after 1 cycles

Sum of electronic and zero-point Energies= -464.842544

Sum of electronic and thermal Energies= -464.829595

Sum of electronic and thermal Enthalpies= -464.828651

Sum of electronic and thermal Free Energies= -464.881901

Dipole moment (field-independent basis, Debye):

X= 0.0001 Y= -0.5709 Z= 0.7081 Tot= 0.9096

****** 1 imaginary frequencies (negative Signs) ******

Frequencies -- -204.4014

- 1. **Data for Dication 5**

Data from file me4bic330++2a-mp2.out

Molecular Weight = 160.26

28

Extracted Standard Orientation:

C 0.807827 1.463103 -0.281478

C -1.364827 0.964894 -0.281165

C -0.450980 1.965952 -0.624747

C 1.364827 -0.964894 -0.281165

C -0.807827 -1.463103 -0.281478

C 0.450980 -1.965952 -0.624747

C -0.749210 -0.171844 0.446673

C 0.749210 0.171844 0.446673

H 1.744904 1.966848 -0.505802

H -2.427840 1.010068 -0.505122

H 2.427840 -1.010068 -0.505122

H -1.744904 -1.966848 -0.505802

C 0.749210 -3.266186 -1.287246

H 1.641980 -3.193216 -1.902908

H -0.086166 -3.589643 -1.902814

H 0.923470 -4.025659 -0.524267

C -0.749210 3.266186 -1.287246

H 0.086166 3.589643 -1.902814

H -0.923470 4.025659 -0.524267

H -1.641980 3.193216 -1.902908

C 1.451596 0.333432 1.837107

H 2.502640 0.574609 1.693840

H 1.369792 -0.596418 2.396345

H 0.972452 1.134844 2.395871

C -1.451596 -0.333432 1.837107

H -1.369792 0.596418 2.396345

H -0.972452 -1.134844 2.395871

H -2.502640 -0.574609 1.693840

Zero-point correction= 0.245514 (Hartree/Particle)

SCF Done: E(RHF) = -463.205634566 A.U. after 1 cycles

Sum of electronic and zero-point Energies= -464.870068

Sum of electronic and thermal Energies= -464.857075

Sum of electronic and thermal Enthalpies= -464.856130

Sum of electronic and thermal Free Energies= -464.908120

Dipole moment (field-independent basis, Debye):

X= -0.0000 Y= 0.0000 Z= -0.7107 Tot= 0.7107

- 1. **Data for TS3**

Data from file ts3a-mp2.log

Molecular Weight = 160.26

28

Extracted Standard Orientation:

C 1.910952 -0.617304 0.054634

C 0.027052 0.486278 0.976549

C 1.440050 0.185926 1.098467

H 2.060264 0.568958 1.904204

C -0.027052 0.486277 -0.976549

C -1.910952 -0.617304 -0.054634

C -1.440050 0.185926 -1.098467

H -2.060264 0.568958 -1.904204

C -0.898483 -0.653693 0.929609

H -0.826296 -1.446619 1.673398

C 0.898482 -0.653694 -0.929609

H 0.826296 -1.446619 -1.673398

C 0.497051 1.797297 -1.528258

H -0.160996 2.618498 -1.253137

H 0.517545 1.721175 -2.618234

H 1.509879 2.000167 -1.187510

C -3.207908 -1.345758 0.001333

H -3.073158 -2.314832 0.480698

H -3.630876 -1.486223 -0.988334

H -3.913726 -0.777597 0.609803

C 3.207908 -1.345758 -0.001333

H 3.630871 -1.486232 0.988335

H 3.913729 -0.777591 -0.609794

H 3.073160 -2.314827 -0.480708

C -0.497050 1.797297 1.528258

H -0.517545 1.721176 2.618234

H -1.509879 2.000167 1.187509

H 0.160996 2.618498 1.253136

Zero-point correction= 0.242287 (Hartree/Particle)

SCF Done: E(RHF) = -463.145583066 A.U. after 1 cycles

Sum of electronic and zero-point Energies= -464.842189

Sum of electronic and thermal Energies= -464.829320

Sum of electronic and thermal Enthalpies= -464.828376

Sum of electronic and thermal Free Energies= -464.880347

Dipole moment (field-independent basis, Debye):

X= 0.0000 Y= -0.5531 Z= 0.0000 Tot= 0.5531

****** 1 imaginary frequencies (negative Signs) ******

Frequencies -- -266.7853

- 1. **Data for Dication 6**

Data from file me4bic330++-mp2.out

Molecular Weight = 160.26

28

Extracted Standard Orientation:

C 1.259467 -1.184913 0.061758

C 1.282919 1.105410 -0.022722

C 1.953982 -0.059861 -0.373272

H 2.900826 -0.086861 -0.895401

C -1.282919 -1.105410 -0.022722

C -1.259467 1.184913 0.061758

C -1.953982 0.059861 -0.373272

H -2.900826 0.086861 -0.895401

C 0.020064 0.762536 0.728626

H 0.080671 1.203498 1.733247

C -0.020064 -0.762536 0.728626

H -0.080671 -1.203498 1.733247

C -1.735209 -2.471063 -0.310607

H -2.788242 -2.500134 -0.577850

H -1.533521 -3.147441 0.520013

H -1.166021 -2.846588 -1.172111

C -1.735209 2.566840 -0.022225

H -2.248647 2.771647 0.930813

H -2.466414 2.704651 -0.815098

H -0.931204 3.294944 -0.092964

C 1.735209 -2.566840 -0.022225

H 2.466414 -2.704651 -0.815098

H 0.931204 -3.294944 -0.092964

H 2.248647 -2.771647 0.930813

C 1.735209 2.471063 -0.310607

H 1.533521 3.147441 0.520013

H 1.166021 2.846588 -1.172111

H 2.788242 2.500134 -0.577850

Zero-point correction= 0.244439 (Hartree/Particle)

SCF Done: E(RHF) = -463.266363724 A.U. after 1 cycles

Sum of electronic and zero-point Energies= -464.921858

Sum of electronic and thermal Energies= -464.908953

Sum of electronic and thermal Enthalpies= -464.908008

Sum of electronic and thermal Free Energies= -464.960124

Dipole moment (field-independent basis, Debye):

X= -0.0000 Y= -0.0000 Z= 0.4830 Tot= 0.4830

1. **DLPNO-CCSD(T)/cc-pVQZ single point energies on all geometries**

| Dication/Level | MP2 | ωB97X-D | M06-2X |
| --- | --- | --- | --- |
| 3 | -465.40398 | -465.40338 | -465.40353 |
| TS1 | -465.36569 | -465.36782 | -465.36238 |
| 7 | -465.37915 | -465.37744 | -465.37717 |
| TS2 | -465.37293 | -465.37249 | -465.37261 |
| 5 | -465.41501 | -465.41484 | -465.41492 |
| TS3 | -465.37401 | -465.37214 | -465.37228 |
| 6 | -465.46870 | -465.46838 | -465.46858 |

1. **Energies (in Hartree) and relative energies (in kcal mol^–1^) at wB97X-D/def2-TZVP//wB97X-D/def2-TZVP**

| Dication/Energy | *E*0+ZPE | *E*0+*E*tot | *E*0+*H*corr | *E*0+*G*corr | Rel *E*0+ZPE | Rel *G*298 |
| --- | --- | --- | --- | --- | --- | --- |
| 3 | -465.93279 | -465.91898 | -465.91803 | -465.97193 | 44.5 | 43.9 |
| TS1 | -465.90398 | -465.89052 | -465.88958 | -465.94336 | 62.6 | 61.8 |
| 7 | -465.91305 | -465.89964 | -465.89870 | -465.95218 | 56.9 | 56.3 |
| TS2 | -465.89972 | -465.88671 | -465.88576 | -465.94044 | 65.3 | 63.7 |
| 5 | -465.94024 | -465.92729 | -465.92635 | -465.97893 | 39.8 | 39.5 |
| TS3 | -465.90029 | -465.88747 | -465.88653 | -465.93840 | 64.9 | 65.0 |
| 6 | -466.00372 | -465.99098 | -465.99004 | -466.04191 | 0.0 | 0.0 |

1. **Geometries at wB97X-D/def2-TZVP**


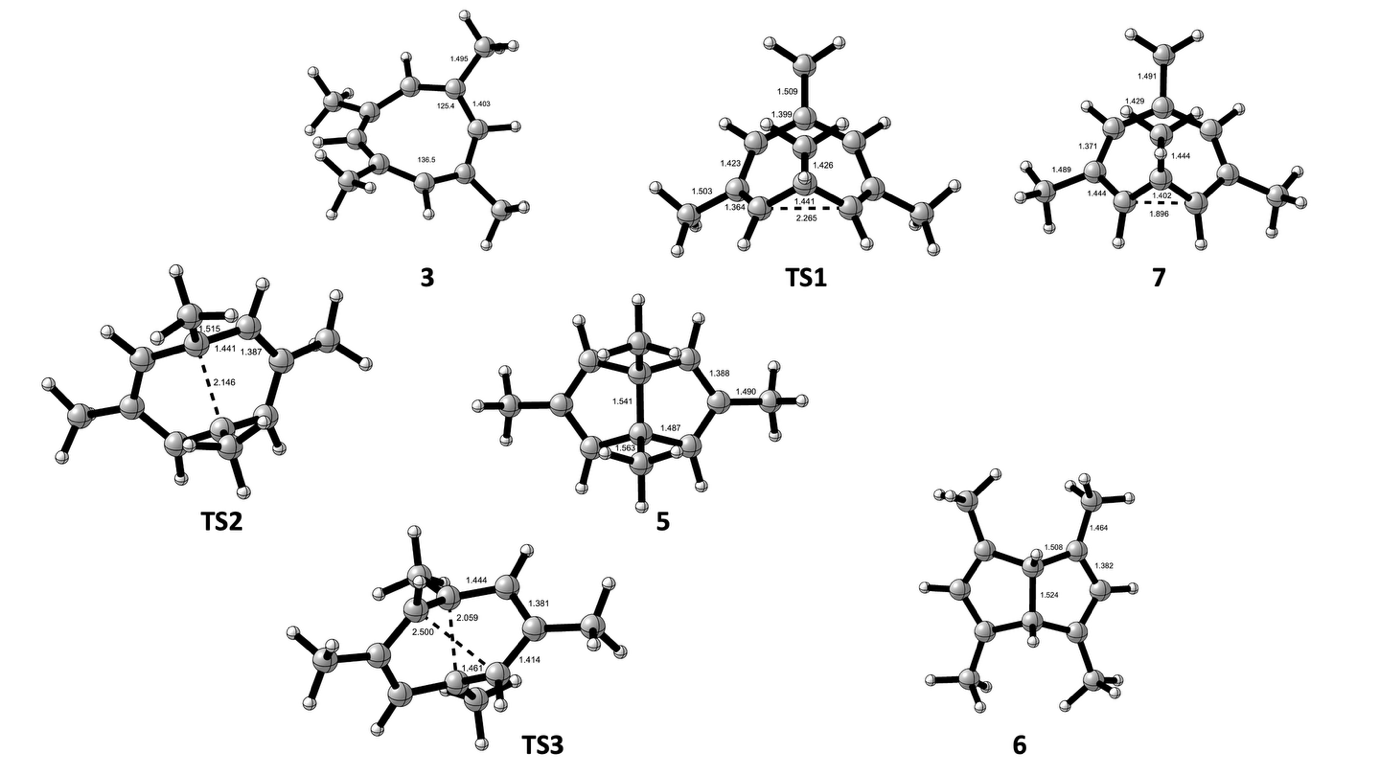

Supplement: Supplementary file 1 — Data S1: Supporting Information. [file JCC-46-0-s001.docx]
